# Duodenal malignant melanoma: Primary and metastatic case series and literature review

**DOI:** 10.1097/MD.0000000000037138

**Published:** 2024-02-09

**Authors:** Bing Zhou, Xiaohua Li, Jincai Liu, Lizi Peng, Xianwei Liu

**Affiliations:** a Department of Pathology, The Second Affiliated Hospital of Jiujiang University, Jiujiang, Jiangxi, China; b Department of General Surgery, The Second Affiliated Hospital of Jiujiang University, Jiujiang, Jiangxi, China; c Department of Gastroenterology, The Second Affiliated Hospital of Jiujiang University, Jiujiang, Jiangxi, China; d Department of Pathology, Jiujiang No. 1 People’s Hospital, Jiujiang, Jiangxi, P.R. China; e Department of General Surgery, Jiujiang No. 1 People’s Hospital, Jiujiang, Jiangxi, P.R. China.

**Keywords:** *BRAF*, clinical pathology, duodenum, malignant melanoma

## Abstract

**Rationale:**

Duodenal malignant melanoma is rare, and its early clinical symptoms are insidious, making it difficult to diagnose in its early stages. Combined with previous literature, We explored the clinicopathological characteristics and v-raf murine sarcoma viral oncogene homolog B1 mutations in primary and metastatic duodenal malignant melanoma, in order to provide some experience on its differential diagnosis and treatment.

**Patient concerns:**

The 2 patients (a 63-year-old female [Patient 1] and a 54-year-old male [Patient 2]) experienced pain and discomfort in their upper abdomen. Additionally, one of them had a history of skin malignant melanoma.

**Diagnoses:**

Patient 1 was diagnosed with primary duodenal malignant melanoma; and Patient 2 was diagnosed with metastatic duodenal malignant melanoma.

**Interventions:**

Patient 1 underwent pancreaticoduodenectomy; and patient 2 underwent complete surgical resection and lymph node dissection.

**Outcomes:**

After surgery, Patient 1 survived after 26 months follow-up, and Patient 2 died of systemic multi-organ circulatory failure after 1 month follow-up.

**Lessons:**

Primary and metastatic cases should be diagnosed through previous medical history analysis and detailed physical and auxiliary examinations. This would enable a diagnosis based on characteristic histomorphology and immunohistochemical markers. An early diagnosis and surgical treatment can prolong patient survival and the molecular inspection of v-raf murine sarcoma viral oncogene homolog B1 mutations can guide follow-up treatment.

## 1. Introduction

Melanoma is a malignant tumor that originates from chromatoblasts and nevus cells, which are both derived from neural crest cells.^[1]^ Malignant melanomas of the digestive tract mucosa can be seen in the rectum, anal canal, and esophagus of patients; those found in the small intestine are mostly due to metastasis.^[2]^ Primary malignant melanoma of the duodenum is extremely rare, with only a small number of cases reported worldwide. Its high degree of malignancy and insidious clinical onset make it exceedingly difficult to diagnose in the early stages; moreover, the prognosis is extremely poor. Combined with previous literature, this article reports on the clinicopathological characteristics and *BRAF* mutations in primary and metastatic duodenal malignant melanoma. This analysis will improve the early diagnosis, early treatment, and risk prognosis assessment of the disease.

## 2. Materials and methods

### 2.1. Materials

**Figure 1:**
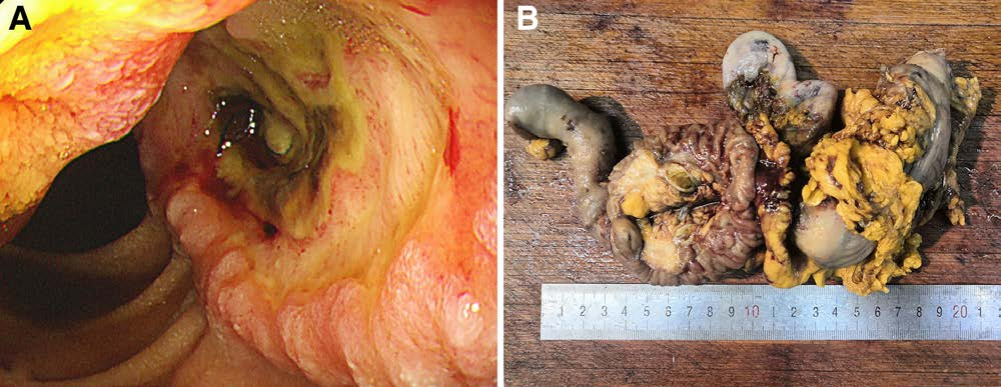
. Endoscopic view and postoperative specimen. (A) Endoscopy displaying a dark brown ulcerative space in the duodenum; (B) the postoperative specimen showing a ulcer-shaped mass at the papilla duodeni major.

Patient 1 was a 63-year-old female. She was admitted to the hospital because of intermittent dull abdominal pain with black stools and weight loss over a 6-month period. She had no history of fever, vomiting, or blood in the stool but had a history of hypertension. Outpatient gastroscopy showed an irregularly shaped mass in the descending part of the duodenum (large papillary ampulla), which occupied three-fourths of the lumen and was fragile with a large amount of necrosis on the surface. Thus, carcinoma of the descending duodenum was considered (Fig. 1A). Biopsies revealed malignant tumors; we considered them poorly differentiated tumors. On physical examination, no lesions or nodules were found in the skin of the whole body; no obvious abnormalities were found upon colonoscopy. The results of routine blood test, stool (1+) analysis, and tumor maker tests were normal and within the reference range.

Patient 2 was a 54-year-old male. He was admitted to the hospital because of persistent upper abdominal pain for one month. He was diagnosed with malignant melanoma of the skin on his neck and chest 2 years prior. He presented with no fever, anorexia, or vomiting. Outpatient gastroscopy revealed a crater and ulcer-type mass in the descending duodenum that had unclear borders and gray-brown scabs on the surface. Thus, we suspected it to be a malignant tumor. The biopsy pathology revealed a malignant tumor, which we diagnosed as a metastatic malignant melanoma. On physical examination, the skin of the whole body was slightly pale and surgical scars were seen on the neck and chest wall; however, there were no obvious enlarged moles or nodules. Blood test results indicated mild anemia, with hemoglobin at 10.0 g/dL; stools (3+) and tumor marker levels were within the reference range.

### 2.2. Materials

Both surgical specimens were incised and fixed with 10% formaldehyde; the next day, they were dehydrated, embedded, and continuously sliced into sections of 4 μm thickness. Next, they underwent conventional hematoxylin and eosin staining. The EnVision method was used for immunohistochemical analysis. Primary antibodies against cytokeratin, vimentin, HMB45, MelanA, S-100, smooth muscle actin, epithelial membrane antigen, CD3, CD20, CK20, CD56, chromogranin, Desmin, Myo-D1, CD117, Dog-1, and Ki67 as well as the EnVision reagent kits (ready-to-use), were purchased from the Fuzhou Maixin Company. Experienced technicians performed standardized operations according to the manufacturer’s instructions.

### 2.3. Materials

Ten of the 8 μm thick sections of the tumor tissues were selected; DNA samples were then extracted using DNA FFPE gene extraction kit (German Qiagen) in strict accordance with the manufacturer’s instructions. DNA purity and concentration were examined using a UV spectrophotometer.We designed the following v-raf murine sarcoma viral oncogene homolog B1 (*BRAF*) exon 15 primers: F: 5′-ACACGCCA AGTCAATCATCC-3′, R: 5′-TCTGGTCCCTCTTGTTCATG-3′. The polymerase chain reaction was performed with 1 μL forward and reverse primers and 1 μL template DNA; the reaction volume was 25 μL. The PCR reaction conditions were as follows: pre-denaturation at 94 °C for 50 seconds, annealing at 56 °C for 30 seconds, and extension at 72 °C for 45 minutes for a total of 35 cycles, followed by a final extension at 72 °C for 10 minutes. The PCR product was purified using a sequencing reaction and an acid enzyme. The gene was sequenced on the ABI 3100 genetic analyzer, and the data obtained were automatically analyzed using the Sequencing Analysis 5.2 software.

## 3. Results

### 3.1. Observation

*Patient 1*: Partial gastric, pancreatic, duodenum, and cholecystectomy specimens were submitted for examination, with section of the greater (10 cm) and lesser (5.5 cm) curvature of the stomach, pancreas (6 × 4 × 4 cm), gall bladder (7.5 × 4 × 3 cm), and duodenum (13 cm in length and 2.5–4 cm in diameter). One 5 × 3cm ulcer-shaped mass was seen at the papilla duodeni major. The cut surface was gray, solid, and slightly hard; it showed penetration of the serous membrane and invasion of the pancreatic tissue (Fig. 1B).

*Patient 2*: A 4 × 3.5 cm ulcerated mass was detected in a section 3 cm from the inferior cut edge of the duodenal tube (10 cm long and 2–5 cm in diameter), occupying three-fourths of the circumference of the intestine; the cut surface was grayish brown, solid, slightly brittle; it showed invasion of the serous membrane.

### 3.2. Microscopic examination

**Figure 2.**
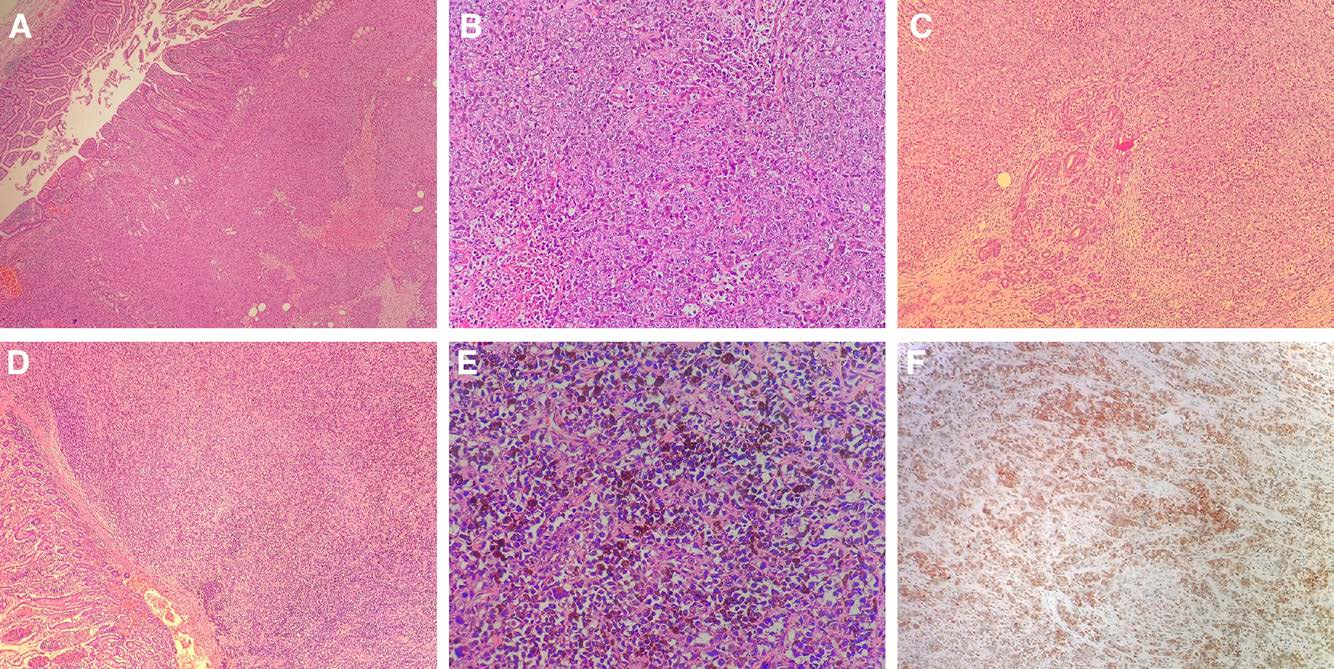
Histopathological features of duodenal malignant melanoma. (A) Tumor-infiltrating growth in the duodenal mucosa and submucosa with no pigmentation (4×); (B) round and oval tumor cells in a flaky array (20×); (C) tumor-invaded pancreatic tissue (4×); (D) tumor-infiltrating growth under the duodenal mucosa with pigmentation (4×); (E) polygonal tumor cells loosely adhered in a pseudo-adenoid array (20×); (F) melan-A (+) using an EnVision method (10×).

*Patient 1*: There was a diffuse infiltrating tumor growth under the duodenal mucosa. Partial mucosal destruction was observed, and no melanin particles were deposited (Fig. 2A). The tumor cells were round and oval in shape with solid nests, patches, and low stromal content. The cytoplasm was eosinophilic, the nucleolus was distinguishable, and mitosis was observable. Inflammation and necrosis were noticed in the background (Fig. 2B). Furthermore, tumor invasion was detected in the pancreas (Fig. 2C).

Patient 2: A diffuse infiltrating tumor growth was observed under the duodenal mucosa; however, here, melanin particles were deposited (Fig. 2D). The tumor cells were epithelioid, polygonal, pseudo-adenoid, and flaky. The cells had a fibrous interstitium and were loosely adhered. Additionally, the cells had a vacuolated cytoplasm as well as deeply stained and irregular nuclei (Fig. 2E). Furthermore, metastasis to a lymph node was observed around the intestine.

### 3.3. Immunohistochemistry

Tumor cells in the 2 cases were positive for vimentin, HMB45, Melan-A (Fig. 2F), and S-100. However, cytokeratin, CK20, CD56, chromogranin, CD20, CD3, CD117, Dog-1, smooth muscle actin, epithelial membrane antigen, Desmin, and Myo-D1 were all negatively expressed. The Ki67 proliferation index ranged from 80% to 90%.

### 3.4. BRAF sequencing

**Figure 3.**
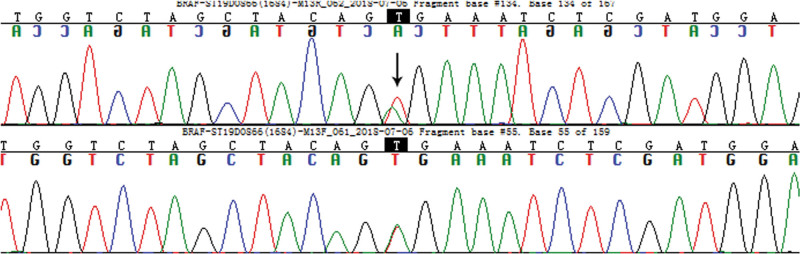
*BRAF* V600E mutation of duodenal malignant melanoma. *BRAF* exon 15 codon mutation 600 (arrow).

Two cases was examined for the *BRAF* V600E mutation. Patient 1 was positive for *BRAF* V600E mutation, the mutation site was T > A at position 1799 of exon 15 (Fig. 3).

### 3.5. Treatment and follow up

Patient 1 underwent resection of the lesion, distal stomach, pancreatic head, and duodenum, supplemented with *BRAF* inhibitor targeted therapy. Patient 2 underwent complete surgical resection, end-to-end anastomosis of the small intestine were performed and lymph node dissection. Both cases were followed up by telephone. Patient 1 died from liver and multiple lymph node metastases across the body 28 months after surgery. Patient 2 died of massive hemorrhage with multiple organ circulation failure 1 month after surgery.

## 4. Discussion

The incidence of malignant melanoma accounts for 1% to 3% of systemic malignant tumors; however, this has been increasing in recent years.^[3]^ Malignant melanoma of the gastrointestinal tract mostly metastasizes from other tissues. This is because of the rich blood supply to the small intestine, enabling metastasis to and from the small intestine. Duodenal metastasis is the most common in the second part of the duodenum and around the ampulla.^[4]^ Patient 2 had a previous malignant melanoma on the chest wall and neck skin. Two years later, tumor metastasis in the descending part of the duodenum was observed alongside the invasion of lymph node.

At present, continuous studies have confirmed that malignant melanoma can occur in the duodenum, which may be related to the migration of embryonic neural crest melanocytes through the umbilical intestine, encapsulation, and cell differentiation with neuroendocrine characteristics of amine uptake decarboxylation.^[5]^ However, because malignant melanoma has a wide range of sites for the ease of systemic metastasis to occur throughout the body, clinical diagnosis of primary duodenal melanoma needs to meet the following criteria: exclusive of concurrent malignant melanoma or precursor lesions at other sites and be a single lesion with no tumor at least 1 year after surgery.^[6,7]^ For Patient 1, after careful inquiry of the previous medical history, there had been no prior melanoma or related lesion resection. Furthermore, after detailed physical examination, videography, and colonoscopy, no obvious lesions or nodules were found. We also summarized 10 cases published between 1990 and 2023 of primary duodenal melanoma (Table 1).^[8–17]^ The male-to-female ratio was 8:3 and patients’ age at diagnosis ranged from 35 to 68 years, with a median of 61 years. Early clinical symptoms are insidious, symptoms such as abdominal pain, dark stools and systemic wasting symptoms (weight loss and anemia) mostly occur in the middle and late stages. The most common site in the second part of duodenum. Due to lacked the specificity for diagnosis by clinical manifestation and endoscopic, only 3 cases could be correctly diagnosed before pathological analysis.

**Table 1:** Summary of the clinicopathological features of primary duodenal melanoma.

Author	Age/sex	Clinical manifestation	Location (duodenum)	Diameter (cm)	Preoperative diagnosis	Pigment	Treatment	Follow-up months	Outcome
Korkolis et al^[8]^	55/M	Abdominal pain, hemorrhage	Second and third part	5.5	Ulcerative tumor	NA	PD	14	AWD
Li et al^[9]^	60/M	Abdominal pain, dark stools	Second part	2.5	Poorly differentiated carcinoma	No	Palliative operation, TCM	46	AWD
Coban et al^[10]^	63/F	Abdominal pain, hematemesis	Second part	5	poorly differentiated neoplasm	Yes	Supportive treatment	3week	DOD
Bendic et al^[11]^	52/M	Weakness, painless jaundice	Vater	1.4	NA	No	PD	4	DOD
Suganuma et al^[12]^	67/M	Anemia	Fourth part	6.2	Malignant melanoma	No	Gastroduodenal-small intestine partial resection	36	AWD
Jain et al^[13]^	35/M	Abdominal pain, vomiting	First,second, and third part	10.4	Malignant melanoma	NA	surgical resection, CT	NA	NA
Kilambi et al^[14]^	35/M	Epigastrium pain	First and second part	13	Malignant melanoma	No	PD, CT	32	AWD
Anvari et al^[15]^	68/M	Fatigue, weakness	Second part	NA	High-grade malignant neoplasm	No	CT	NA	DOD
Surjan et al^[16]^	40/F	Abdominal pain, anorexia	Second, third and fourth part	18	Gastrointestinal stromal tumor	Yes	PD	36	AWD
Nguyen et al^[17]^	61/M	Anemia, fatigue	Third part	10.5	Poorly differentiated neoplastic	NA	RT + IT	38	AWD
Our case	63/F	Abdominal pain, dark stools	Second part	4	Poorly differentiated neoplastic	No	PD + TT	28	DOD

AWD = alive without disease; CT = chemotherapy; DOD = dead of disease; F = female; IT = immunotherapy; M = male; NA = data not available; PD = pancreaticoduodenectomy; RT = radiotherapy; TCM = traditional Chinese medicine; TT = targeted therapy.

Tumors under endoscopy are often large with an ulcer-like or polypoid appearance. The surface of the tumors appears to be gray-black, which is helpful for correct diagnosis. In Patient 2, the tumors showed pigment deposits, which provided clues for a clear diagnosis. Primary malignant duodenal melanoma was found through literature review the no pigments-to-pigments ratio was 3:1, which makes diagnosis difficult. Thus, it must be distinguished from poorly differentiated tumors, lymphoma, neuroendocrine tumors, and stromal tumors of the duodenum. The structure of duodenal malignant melanoma under a microscope is similar to that of malignant melanoma of other body parts. Tumor cells seen under the mucosa have round, oval, or polygonal diffuse sheet-like morphologies and growth; furthermore, in some areas, tumor cells have poor adhesion and are loosely arranged, with large nuclei and obvious nucleoli. The diagnosis of melanoma additionally requires characteristic immunohistochemical markers of melanoma. Among them, HMB45 and Melan-A have poor sensitivity but good specificity whereas S-100 has strong sensitivity but poor specificity; thus, their combined application can improve the diagnosis rate. Here, both patients expressed the above immunological markers. Meanwhile, the Ki67 proliferation index was high, indicating a poor prognosis. Under an electron microscope, melanin bodies at different developmental stages can be seen in the cytoplasm of tumor cells, which are of great significance for the diagnosis of duodenal malignant melanoma.^[18]^

Although it is difficult to differentiate between primary and metastatic malignant melanoma of the duodenal, except for the past medical history, some studies are suggestive. It has been found that primary melanoma of the small intestine is usually a single lesion with intestinal bleeding and progressive obstruction, whereas metastatic cases are more common with multiple lesions and often have acute abdominal symptoms caused by intestinal entrapment or intestinal obstruction as the first symptom.^[19,20]^ Morphologically, since more than 95% of malignant melanoma occur in the skin and eye, and pigment is seen more often in metastatic cases.^[21]^ In Patient 1, with longer digestive symptoms and no pigments deposition, similar to the above study. At the same time, the author believes that if there is a precursor lesion or an abnormal proliferation of melanoma in the duodenum, the primary melanoma is more likely to be present with the exception of previous history. Unfortunately, the disease was in the progressive stage in both cases and no early biopsy data were obtained.

Recently, molecular genetic analysis has shown that malignant melanoma is closely related to *BRAF. BRAF* can activate the MEK signal transduction pathway through the phosphorylation and activation of MEK to promote the proliferation of melanoma cells and inhibit their apoptosis. This confers a poor clinical outcome for the patients.^[22]^ The *BRAF* mutation rate is as high as 60% in Caucasians. Although Asians do not have a mutation rate as high as Caucasians, nearly one-third of melanoma patients still have *BRAF*-sensitive site mutations. Of these, exon 15 mutation V600 is accountable for nearly 90%. Moreover, *BRAF* mutation is regarded as a target for tyrosine kinase inhibitor intervention in signal transduction pathway therapy, with which significant progress has been made in the treatment of malignant melanoma.^[23]^ Therefore, the 2019 Chinese society of clinical oncology Melanoma Diagnosis and Treatment Guidelines recommend the routine detection of *BRAF* mutations in malignant melanoma patients, improving the significance of subsequent treatment decisions. In our study, Patient 1 had a T > A mutation at position 1799 of exon 15 in *BRAF* V600E.

Patients with malignant melanoma originating in the mucosa have worse prognosis than those with malignant melanoma occurring in the skin. Furthermore, malignant melanoma originating in the mucosa is marginally sensitive to radiotherapy and chemotherapy; therefore, complete surgical resection is the first choice for treatment. For patients with solitary duodenal metastasis or obvious intestinal obstructions, surgical resection can effectively relieve symptoms and prolong survival. Nevertheless, the prognosis of metastatic patients is still extremely poor, with a median survival time of about 7 months.^[24]^ Patient 2 underwent complete surgical resection and lymph node dissection, 1 month after surgery, the patient died of systemic multi-organ circulatory failure. The prognosis of primary cases is slightly better than that of metastatic cases. The median survival time after surgery is about 28 months, and later deaths are mostly caused by distant metastases to the liver, brain, and other organs.^[14,17]^ Patient 1 underwent pancreaticoduodenectomy, supplemented with *BRAF* inhibitor targeted therapy. This patient survived 26 months after surgery, which was consistent with reports in the literature.

To the best of our knowledge, this is the first comparative case report on the clinicopathological features of primary and metastatic duodenal malignant melanoma. We believe that our study makes a significant contribution to the literature, not only contributing to a better understanding of the diagnosis and treatment of this rare case, but also provide some experience for identifying the histological source and primary site of duodenal malignant melanoma. However, our study has some limitations including the total number of patients is relatively small and retrospective design. Moreover, we did not perform a multivariate analysis due to the limited available data. In the future, it is necessary for more patients and molecular alterations are expected to be analyzed.

## 5. Conclusions

In conclusion, malignant melanoma of the duodenum is extremely rare and has atypical clinical symptoms. Previous medical history and a detailed physical and auxiliary examination are required for effective diagnosis of metastatic or primary cases. Diagnosis depends on characteristic histological morphology and immunohistochemical markers. Although the prognosis of patients with primary tumors is slightly better than that of patients with metastatic tumors, the overall prognosis is still poor. However, early examination, early diagnosis, and early surgical treatment can prolong survival. Molecular examination of *BRAF* mutations can help in subsequent treatments.

## Author contributions

**Conceptualization:** Bing Zhou, Xian Wei Liu.

**Data curation:** Bing Zhou, Xiaohua Li, Jincai Liu, Lizi Peng, Xian Wei Liu.

**Methodology:** Xiaohua Li, Jincai Liu.

**Validation:** Lizi Peng.

**Writing – original draft:** Bing Zhou.

**Writing – review & editing:** Bing Zhou, Xian Wei Liu.
